# Current Characterization Techniques Applied to Microalgae–Fungal Pellets: Unraveling the Mechanisms of Adhesion and Stability Focused on Nutrient Recovery/Recycling and Bioprocess Diversification

**DOI:** 10.3390/biotech15030049

**Published:** 2026-06-29

**Authors:** João Victor Oliveira Nascimento da Silva, Carlos Eduardo de Farias Silva, Tomás Agustín Rearte, Eleni Kougia, Giorgos Markou, Albanise Enide da Silva

**Affiliations:** 1 Technology Center, Federal University of Alagoas, Maceió 57072-970, Brazil; 2 Cátedra de Química Inorgánica y Analítica, Facultad de Agronomía, Universidad de Buenos Aires, Buenos Aires C1417DSE, Argentina; 3 Consejo Nacional de Investigaciones Científicas y Técnicas (CONICET), Buenos Aires C1425FQB, Argentina; 4 Institute of Technology of Agricultural Products, ELGO-Dimitra, 14123 Athens, Greece

**Keywords:** co-aggregation, microalgae–fungal cultivation, microalga–fungus, microalga, filamentous fungus, microalga-fungus consortium, microalgae-fungi

## Abstract

Microalgae–fungal pellets have been studied as a versatile and robust biotechnological platform, offering significant advantages for microalgal biomass harvesting, wastewater treatment, biofuels production and/or obtaining of value-added products. This review presents an integrated analysis of the mechanisms governing the formation, stability, and functionality of these systems, combining physicochemical, biological, and mathematical modelling approaches and aims to describe the current state of the art and main research needs. The aggregation process is strongly influenced by the complementarity of the surface properties of microalgae and filamentous fungi, including electrostatic interactions, production of extracellular polymeric substances (EPSs), and modifications in surface roughness. Recent advances in multiscale characterization techniques, such as confocal microscopy, micro-computed tomography, atomic force microscopy, and X-ray photoelectron spectroscopy, have allowed a more precise elucidation of the internal architecture and surface chemistry of the pellets. In parallel, biological characterization through enzymatic assays, oxidative stress biomarkers, and photosynthetic activity analyses has provided relevant information on the metabolic responses and functional resilience of the consortium. Additionally, the incorporation of mathematical flocculation models can contribute to the prediction of pellet growth, density, and stability, supporting process optimization and application. The understanding of these interaction phenomena is important for the design of high-yield and efficient systems, including their development and validation, to expand the use of microalgae–fungal pellets in bioprocesses, as evidenced by this review.

## 1. Introduction

Microalgae have expanded their application possibilities, especially in the production of biofuels, biofertilizers, and biosorbents as well as the bioremediation of urban and agro-industrial wastewaters. The economic feasibility of commercial microalgae-based industries is currently hindered by the substantial operational and capital expenditures linked to the harvesting and dewatering stages, which represent a critical energy and cost bottleneck in the overall biomass production process [1,2]. This limitation is fundamentally driven by the technical challenges of isolating cells from a dilute medium (<1 g·L^−1^) due to their small size (<30 µm) and the electro-negative properties of their cell membranes, which require energy-intensive recovery processes that often exceed the energy value of the biomass itself [3].

The association between microalgae and filamentous fungi has emerged as an environmentally viable technology to reduce biomass harvesting costs [4,5,6]. In this context, fungal-mediated pelletization has attracted considerable attention due to the properties of the mycelial matrix, which promote electrostatic attraction between positively charged hyphae and negatively charged microalgal cells, as well as the production of extracellular polymeric substances (EPSs), responsible for pellet aggregation and stability [6,7,8]. Most studies have focused on wastewater treatment, in which granule adhesion and stability are essential for long-term operational performance [7,9]. Among microalgae, diatoms (*Ochrophyta*) are distinguished by their siliceous frustules, which provide a high specific surface area that enhances heavy metal adsorption, in addition to their high photosynthetic efficiency and rapid cellular growth [10,11].

Likewise, species belonging to the phylum *Chlorophyta*, particularly the genera *Chlorella*, *Monoraphidium*, and *Scenedesmus*, have been extensively investigated in treatment systems due to their physiological robustness and high contaminant removal efficiency [12,13]. Beyond their environmental remediation potential, microalgae represent a promising platform for the production of high-value bioproducts owing to their rapid growth rate, elevated photosynthetic and CO_2_ fixation capacities, and their ability to accumulate lipids, carbohydrates, and bioactive compounds [14,15]. Although the conversion of microalgal biomass into biodiesel and bioethanol still faces techno-economic constraints, biogas production and the development of microalgae-based agricultural products have emerged as more feasible and sustainable alternatives [16,17].

According to Li et al. (2022) [18], the biological activity and cell surface composition of microalgae directly influence their physicochemical properties, particularly surface charge. Variations in structural components such as scales, membranes, and glycoprotein-rich cell walls can result in zeta potential (ζ) values ranging from −2.39 to −43.2 mV among green microalgae. This predominantly negative surface charge is mainly attributed to the presence of carboxyl and amino functional groups and is further affected by environmental conditions, including pH, inorganic salt concentration, growth stage, and microalgal species [18]. In contrast, fungal cells generally exhibit a positive surface potential due to the composition of their cell walls, which consist of fibrillar components such as chitin, β-(1→3)- and β-(1→6)-glucans, and chitosan, embedded within a matrix containing α-(1→3)-glucan, galactomannan proteins, xylomannan proteins, polyglucuronic acid, glucuronomannan proteins, and β-glucans [6,19]. Additionally, fungi are recognized for their ability to secrete substantial amounts of extracellular polymeric substances (EPSs), composed primarily of polysaccharides and proteins, along with lipids, nucleic acids, and humic-like substances derived from organic matter degradation [4,9].

These contrasting surface properties create favorable conditions for electrostatic attraction between microalgal and fungal cells, making zeta potential a key parameter for understanding pellet formation and stability in co-culture systems. In general, absolute zeta potential values above ±30 mV promote colloidal stability by increasing repulsive forces between particles, whereas lower absolute values reduce electrostatic repulsion and facilitate cell-to-cell interactions, aggregation, flocculation, and ultimately pellet formation through the predominance of attractive forces. However, it is important to emphasize that zeta potential does not act as an isolated factor in the pelletization process; rather, its effect is coupled with other operational parameters, such as pH, presence of ions (mainly Na^+^, Ca^2+^, NO_3_^−^ and HPO_4_^2−^), and, more importantly, chemical adsorption mechanisms associated with interactions among surface functional groups, which collectively govern the dynamics of pellet formation [6,7].

As a result, fungal biomass has been widely explored in strategic industrial applications, ranging from the production of biofuels, with particular emphasis on genera such as *Aspergillus*, *Trichoderma* and *Penicillium* [20], to obtaining enzymes of biotechnological interest, such as xylanases, cellulases, proteases and lipases [21]. Additionally, this biomass type represents a promising source for the generation of high value-added products, including structural biopolymers such as chitosan and glucans, as well as proteins with potential applications in the food, pharmaceutical, and material sectors [22]. From a circular economy perspective, the use of microalgal and fungal biomass could contribute to the valorization of residual waste streams and the reduction of dependence on fossil raw materials, aligning with the principles of maximizing the value of materials throughout their life cycle [23].

In general, this association is characterized as symbiotic because it confers benefits to the mutual growth of the strains involved, resulting from ecological interactions established between microalgae and fungi (through chemical or physical mechanisms). Microalgae play a central role in fixing carbon dioxide (CO_2_) and producing organic compounds that serve as a source of carbon and energy for fungal growth. In turn, filamentous fungi benefit microalgae through structural protection and facilitation of mineral nutrient absorption, creating a more stable microenvironment that is favorable for algal development [4,24].

Recent advances have improved the understanding of microalgae–fungal pellets, encouraging the use of characterization techniques beyond conventional morphological analyses. Spectroscopic, surface, imaging, and mechanical methods have provided deeper insights into pellet formation, stability, and performance, particularly regarding electrostatic interactions, extracellular polymeric substances (EPSs), and metabolic responses [25,26,27].

At the same time, DLVO/XDLVO-based models have become important tools for predicting microalgae–fungal aggregation by describing the physicochemical forces involved in pellet formation and stability. Combined with biological assays, these approaches help relate structural and thermodynamic properties to the physiological responses of microbial consortia under different environmental conditions [28,29,30].

This integrated approach reinforces the interpretation of microalgae–fungal pellets as complex, dynamic, and multifunctional biostructured systems. Given this conceptual and methodological advancement, it becomes essential to critically discuss the characterization techniques currently applied to these systems, highlighting their potential, limitations, and prospects for future applications in biotechnological processes, the central objective of this critical review.

## 2. Applications of Microalgae–Fungal Pellets

**Figure 1:**
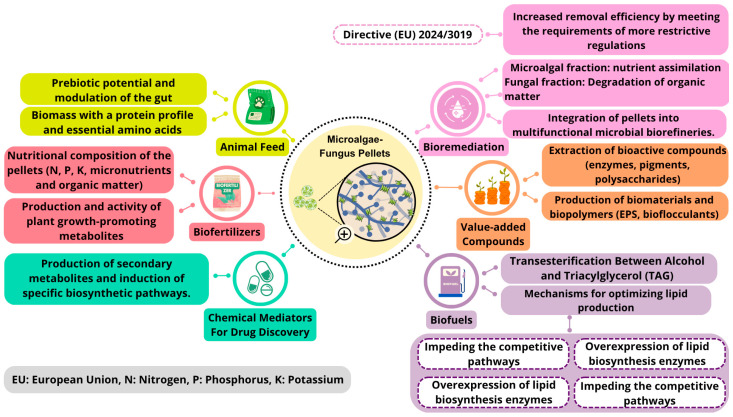
Current applications and future perspectives of microalgae–fungal pellets for wastewater treatment, biofuel production, resource recovery, and high-value bioproduct generation.

Pellets composed of microalgal and fungal matrices exhibit high biotechnological versatility, with potential applications in bioenergy pathways, bioremediation processes, agricultural formulations, and pharmaceutical systems (Figure 1). The synergistic combination of microalgae and fungi could potentially contribute to operational gains, which might include improved structural stability, enhanced combined metabolic efficiency, and possible reductions in costs associated with critical industrial steps, particularly harvesting, solid–liquid separation, and biomass recovery. Compared to traditional monocultures or co-cultures, such as microalgae–bacteria, the microalgae–fungal configuration offers superior performance due to the robustness of the fungal matrix, surface polarity, and high adsorption and cell aggregation capacity. These properties make this type of pelletization a strategic platform for the development and optimization of advanced biotechnological processes [31,32].

The shortcomings of conventional wastewater treatment systems based on microalgae–bacteria symbiosis are reflected in their dependence on metabolic interactions and key molecules (vitamins and quorum-sensing signals) to achieve feasible removal efficiencies [33]. This characteristic highlights the application of microalgae–fungal pellets, since, in conventional wastewaters treatment systems, the chemical composition is highly variable, with abrupt fluctuations in organic load, and the intermittent presence of metals can destabilize these interactions, interrupting the flow of signaling molecules and shifting the relationship from mutualism to competition or even antagonism between microalgae and bacteria [34,35,36].

In this context, microalgae–fungal pellets have shown promising results for wastewater treatment (Table 1), potentially enhancing contact with the pollutant load and promoting the formation of internal microenvironments that may help buffer variations in composition and toxicity [33]. The results presented in Table 1 demonstrate that the removal efficiencies of organic matter, nitrogen, and phosphorus by microalgae–fungal pellets can vary substantially among different studies. These variations are associated not only with the characteristics of the wastewater being treated but also with the operational conditions employed during consortium cultivation.

Factors such as light intensity, photoperiod, aeration rate, inorganic carbon availability, temperature, pH, and the C:N:P (or COD:N:P) ratio directly influence microalgal and fungal growth, as well as nutrient assimilation and organic matter degradation processes. In this context, maintaining a balanced carbon, nitrogen, and phosphorus ratio is essential for sustaining microbial metabolism and ensuring efficient nutrient removal, since nutritional imbalances can impair biological performance and lead to the accumulation of residual carbon within the system [37,38,39].

Furthermore, the composition of the microbial consortium itself plays a decisive role in process performance, as different species exhibit distinct physiological, metabolic, and morphological characteristics, resulting in variations in cell aggregation, pellet formation, mass transfer, and nutrient-utilization capacities. Therefore, the combined effects of operational conditions and the intrinsic characteristics of each microalgae–fungal inoculum contribute to the wide range of removal efficiencies reported in the literature, highlighting the need for system-specific optimization according to the intended application and wastewater type [40,41].

**Table 1:** Studies on the application of microalgae–fungal pellets in wastewater treatment.

Microbial Consortium	Wastewater and Initial Characterization	% Removal			References
		COD	TN	TP	
*Chlorella pyrenoidosa Aspergillus oryzae*	Simulated Potato Starch Wastewater (COD: 12266.82 ± 754.26 mg·L^−1^; TN: 611.30 ± 1.78 mg·L^−1^; TP: 49.59 ± 1.45 mg·L^−1^)	92.08	83.56	96.58	[42]
*Chlorella sorokiniana Aspergillus oryzae*	Artificial swine wastewater (COD: 1000 mg·L^−1^; TP: 10.6 mg·L^−1^)	85.90	-	60.30	[33]
*Chlorella sorokiniana Aspergillus oryzae*	Wastewater from the fish filleting industry (COD: 330 mg·L^−1^; TN: 43 mg·L^−1^; TP: 5.3 mg·L^−1^)	69.4	93.4	92.9	[43]
*Chlorella vulgaris Aspergillus oryzae*	Artificial wastewater (COD: 866.67 ± 47 mg·L^−1^; TN: 1094.43 ± 0.55 mg·L^−1^; TP: 21.67 ± 0.21 mg·L^−1^)	74.53	56.00	98.55	[44]
*Chlorella vulgaris Ganoderma lucidum*	Biogas wastewater (COD: 1106.53 ± 21.92 mg·L^−1^; TN: 213.56 ± 14.27 mg·L^−1^; TP: 20.08 ± 2.15 mg·L^−1^)	92.17	89.83	90.31	[45]
*Chlorella vulgaris Ganoderma lucidum*	Anaerobically digested swine wastewater (COD: 289.98 ± 11.05 mg·L^−1^; TN: 51.84 ± 4.76 mg·L^−1^; TP: 12.86 ± 2.05 mg·L^−1^)	79.74	74.28	85.37	[46]
*Scenedesmus* sp. *Aspergillus tubingensis*	Effluent from a seafood processing plant (COD: 4097 ± 695 mg·L^−1^; TN: 218.4 ± 0.0 mg·L^−1^; TP: 32.0 ± 2.9 mg·L^−1^)	92.69	80.39	75.48	[47]
*Chlorella* sp. *Aspergillus niger*	Wastewater from industrial fish farming (COD: 182.5 ± 2.5 mg·L^−1^; TN: 14.5 ± 0.5 mg·L^−1^; TP: 1.895 ± 0.025 mg·L^−1^)	84.19	89.50	93.46	[7]
*Chlorella vulgaris Penicillium* sp.	Wastewater from soy sauce production (COD: 4602.7 mg·L^−1^; TN: 178.2 mg·L^−1^; TP: 26.0 mg·L^−1^)	78.80	82.83	93.07	[48]
*Navicula seminulum Cordyceps militaris*	Aquaculture wastewater (COD: 318.63 ± 10.45 mg·L^−1^; TN: 51.89 ± 2.16 mg·L^−1^; TP: 8.64 ± 0.35 mg·L^−1^)	52.15	63.03	63.19	[49]

COD—Chemical Oxygen Demand, TN—Total Nitrogen, and TP—Total Phosphorus.

Due to the competitive advantages shown by these microorganisms in converting organic and inorganic compounds, such as nitrogen and phosphorus (nutrients that contribute to eutrophication), into biomass for the production of valuable and marketable bioproducts, the inclusion of microalgae and fungi in waste treatment for secondary and tertiary wastewaters has gained significant prominence from both a scientific and commercial perspective [4]. As a recent example, Silva et al. (2025) [50] demonstrated high efficiency in the simultaneous removal of nutrients and organic matter from soft drink industry wastewater in reactors with moderate aeration (1.5 vvm), achieving removal efficiencies of 92.5% COD, 79.3% total nitrogen and 83.4% total phosphorus (initial concentrations of 172.86 ± 2.42 mg·L^−1^, 14.51 ± 0.05 mg·L^−1^ and 6.18 ± 0.02 mg·L^−1^, respectively) after 2.5 h of operation, indicating high treatment efficiency associated with the formation of *Tetradesmus obliquus* and *Chlorella* sp. pellets associated with the *Penicillium gravinicasei* fungus.

The biochemical and physiological mechanisms involved in the removal of suspended solids and nutrients in microalgae–fungal pellets systems are based on the conversion of the particulate fraction present in the wastewater into soluble nutrients through the action of extracellular enzymes released by the fungus, allowing the heterotrophic assimilation of these solids by the fungal biomass, as well as fundamental physicochemical interactions for the formation and stability of the pellets, including electrostatic neutralization, adhesion mediated by exopolysaccharides, and protein–surface interactions, which favor the encapsulation and retention of the colloidal suspended particulate matter and microalgae by the mycelium. The microalga, in turn, plays an essential complementary role through photosynthesis, generating oxygen and assimilating dissolved nutrients through mechanisms such as vacuolar sequestration, ion exchange, complexation, and the action of metallothioneins (cysteine-rich proteins) [51].

The production of value-added compounds (lipids, proteins, and complex molecules), as well as biofuels from microalgae–fungal biomass, has been discussed given some characteristics, for example, the possibility of achieving lipid contents exceeding 20% in filamentous fungi *Aspergillus tubingensis* NRRL 4700, *Cunninghamella echinulata* NRRL 3655, *Mortierella isabellina* NRRL 1757, *Mucor circinelloides* NRRL 3631, *Mucor miehei* NRRL 3169 and *Mucor racemosus* UCD 71-20 in a glycerol-based medium [52], percentages also achieved in specimens such as *Absidia cylindrospora*, *Lichtheimia brasiliensis*, *Circinella muscae*, *Syncephalastrum racemosum*, *Rhizopus arrhizus* var. *arrhizus* and *Rhizopus stolonifera* cultivated in synthetic medium [53].

Furthermore, the integration of filamentous fungi-assisted harvesting strategies into microalgae biorefinery platforms represents a significant ecological advancement with a sharp decrease in waste generation, as well as an economically viable alternative, helping to overcome the biggest challenge in implementing microalgae-based biofuel generation through increased biomass capture efficiency, better control of operational parameters, and the possibility of cultivation in wastewaters to reduce operational costs [54].

The interaction occurs when positively charged fungal cells act as neutralizing agents, compensating for the predominant negative charge on the surfaces of microalgal cells. This electrostatic interaction promotes cell-to-cell adhesion, leading to the aggregation and retention of microalgae. As evidence of the high potential of microalgal harvesting via pelletization using filamentous fungi, efficiencies of 80–85% have been reported for an algal-to-fungal biomass ratio of approximately 1:1 (*Chlamydomonas reinhardtii* and *Mortierella alpina*) [55], as well as values exceeding 94% for a 1:2 ratio (*Scenedesmus* sp. and *Trichoderma reesei*) [56].

Studies also emphasize that integrating this harvesting approach with subsequent routes for lipid extraction, catalytic conversion, and advanced fuel production substantially improves the overall efficiency of the biorefinery, since microalgae–fungal pellets have higher density, recoverable biomass efficiency, and compatibility with biochemical processes such as transesterification and anaerobic digestion [45,57].

Additionally, studies involving seventeen fungal species demonstrated that the physicochemical properties of biodiesel, including kinematic viscosity, density, saponification index, iodine value, cetane index, oxidative stability, and higher heating value, are closely related to the fatty acid composition of the lipid feedstock. Overall, the evaluated fungal lipids produced biodiesel with characteristics that met or approached international quality standards, reinforcing the potential of oleaginous fungi as promising feedstocks for sustainable biofuel production [53].

As shown in Table 2, biodiesel produced from microalgal and fungal biomass generally exhibits fuel properties comparable to those obtained from conventional vegetable oils and, in several cases, approaches the quality specifications established by international standards. Microalgal species, including *Nannochloropsis* sp., *Chlorella homosphaera*, *Chlorella protothecoides*, and *Spirulina*, have demonstrated favorable viscosity, density, and cetane index values, reflecting the suitability of their lipid profiles for biodiesel production. Similarly, biodiesel derived from fungal biomass, particularly Mucorales species [53], has shown promising fuel characteristics, highlighting the potential of oleaginous fungi as alternative lipid sources. However, differences in oxidative stability, iodine value, and heating value among feedstocks indicate that biomass composition and fatty acid profiles remain key factors governing biodiesel quality. Overall, these findings reinforce the potential of microalgae and fungi as sustainable and versatile feedstocks for the production of renewable biofuels, while emphasizing the importance of selecting suitable microbial strains according to the desired fuel properties.

The symbiotic integration between microalgae and fungi can result in biomass with biochemical profiles more favorable for lipid production, which is fundamental for biodiesel synthesis. This is due to the stimulation of metabolic pathways related to the stress imposed on the generated pellet, the accumulation of triglycerides or carbohydrates, and the secretion of exopolysaccharides, increasing the energy value of the biomass. Furthermore, pellets can be directly applied in thermochemical routes, such as pyrolysis or gasification, or in biochemical routes, such as carbohydrate fermentation. The combination of lipid-rich microalgae with filamentous fungi reinforces the potential of these consortia for obtaining liquid (biodiesel, bio-oil) and solid (biochar) biofuels [51].

Regarding biomethane production, evidence regarding the application of *Aspergillus lentulus* fungal granules in the harvesting (and consequently pelletization) process of the microalgae *Chroococcus* in a 1:3 ratio not only resulted in capture efficiencies close to 100% in just 6 h of incubation (without the need for a flocculant fraction) but also in significant increases in digestibility (>54%) and biomethane production (>50%) compared to the application of biomass from an algal monoculture. Furthermore, it was possible to obtain soluble sugars (≈360 mg·L^−1^), indicating that the symbiotic interaction promoted the intensification of the release and/or transformation of structural and extracellular carbohydrates via the hydrolytic action of fungal enzymes [71].

Applications of microalgal and fungal biomass in animal feed production are also supported by multiple studies that focus on reusing residual biomass generated as byproducts of industrial processes, such as wastewater treatment or fermentation systems, in which these microorganisms are cultivated. The use of fungal biomass as an alternative protein ingredient has gained prominence due to its high nutritional density, biotechnological versatility, and potential to reduce dependence on traditional sources such as fishmeal, soy protein concentrate, and wheat gluten. In this context, *Paecilomyces variotii* has emerged as a promising species, especially in aquaculture production systems [72].

The study by Hooft et al. (2024) [72] demonstrated that the biomass of this fungus presented a robust nutritional profile, characterized by high levels of crude protein (625.1 g·kg^−1^ dry matter), significant concentrations of essential amino acids such as lysine, leucine, isoleucine, and valine (196.7 g·kg^−1^ dry matter) and substantial levels of non-essential amino acids, including glutamic acid, aspartic acid, alanine, and proline (181.5 g·kg^−1^ dry matter). Furthermore, the presence of total minerals such as phosphorus, calcium, and sulfur (total of 44.2 g·kg^−1^ dry matter) reinforces its value as a functional ingredient [72]. The incorporation of this biomass in proportions of 5 to 20% in diets formulated with soy protein concentrate and wheat gluten for Atlantic salmon resulted in significant improvements in feed conversion rate, indicating greater efficiency in nutrient utilization. Furthermore, regulation of cytokines, effector molecules, and transcription factors such as *irf4* was evidenced, highlighting that the fungal biomass not only partially replaces conventional protein sources but also contributes to strengthening the animals’ immune system. These results reinforce the viability of using *P. variotii* as a sustainable and high-performance protein substitute, aligned with innovation trends in aquaculture nutrition and the principles of the circular economy [72].

Relevant aspects are related to the reuse of microalgal biomass for animal feed formulation. As demonstrated by Wang et al. (2025) [73], the use of a microalgal consortium cultivated in anaerobic digestate from mesophilic wastewater treatment requires attention in post-harvest processing. The authors observed that biomass previously washed with deionized water had inferior results compared to unwashed biomass (44%), suggesting that the presence of residual digestate components may establish a beneficial association between *Moina* and microalgae, contributing to better growth rates. However, the use of microalgal biomass from anaerobic sludge may favor the growth of toxic cyanobacteria, resulting in negative effects.

The understanding of the functional mechanisms of edible fungal polysaccharides has evolved from a bioactivity-centered perspective toward a more integrated framework encompassing interfacial interactions, gut microbiota modulation, and specific molecular pathways. Recent studies have demonstrated that these biopolymers exert their biological effects through multiple mechanisms, including modulation of the gut microbiota, production of short-chain fatty acids (SCFAs), interaction with pattern recognition receptors (PRRs), activation of signaling pathways such as NF-κB, MAPK, Nrf2, and PI3K/Akt, as well as the regulation of inflammatory, immunological, and metabolic processes. Furthermore, emerging evidences highlight the intestinal mucus layer as a critical biological interface mediating the activity of these compounds, since interactions between polysaccharides and mucins can influence their diffusion, bioavailability, and physiological functionality. Consequently, the functional effects of fungal polysaccharides depend not only on their chemical composition but also on the interactions established at the polysaccharide–mucus–microbiota interface, which play a pivotal role in modulating intestinal barrier integrity and host responses [74,75].

Despite the promising results achieved under controlled laboratory conditions, large-scale implementation of microalgae–fungal systems remains challenging due to fluctuations in biomass productivity, high susceptibility to microbial contamination, and the requirement for complex operational infrastructure [76]. The performance of these consortia is strongly influenced by environmental and operational factors, including temperature, light intensity, pH, and aeration rate, which directly affect cell growth, nutrient uptake, pellet formation and stability, as well as the biochemical composition of the resulting biomass. Among these parameters, temperature plays a critical role by modulating cellular metabolism and altering the relative abundance of biomacromolecules, particularly proteins, through denaturation processes and the disruption of non-covalent bonds [77]. Likewise, light intensity directly influences the photosynthetic activity of microalgae and, consequently, the metabolic interactions established between microalgal and fungal partners within the consortium [78].

Although harvesting technologies have achieved high biomass recovery efficiencies, the variability and magnitude of associated costs continue to limit the economic competitiveness of pathways focused exclusively on biofuel production. In contrast, alternative valorization strategies, such as biofertilizer production and the coproduction of biogas and biocrude, have demonstrated greater economic resilience, exhibiting superior internal rate of return (IRR) and net present value (NPV) indicators, even under scenarios involving substantial capital investment [79].

## 3. Pellet Formation Morphology and the Influence of Operating Parameters

**Figure 2:**
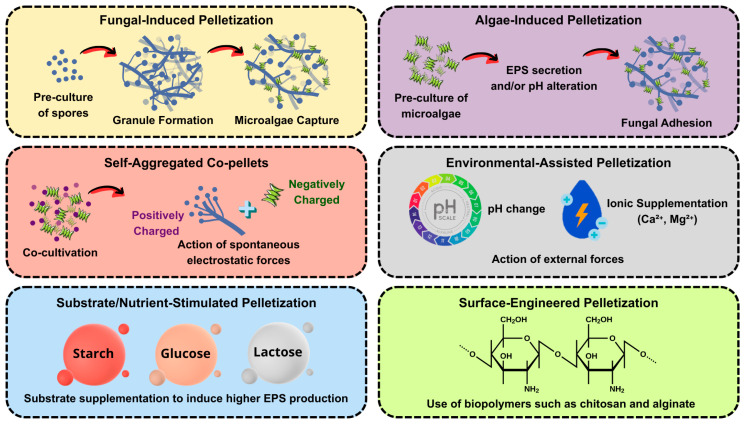
Mechanisms of pellet formation in microalgae–fungal systems under co-cultivation conditions and/or physicochemical stimuli.

The study of different methodologies for the formation of microalgae–fungal pellets is essential for optimizing and solving problems inherent to their cultivation, as well as allowing greater flexibility in the adoption of this biological mechanism in different industrial sectors. Methods involving only the target microorganisms, such as microalgal or fungal induction and spontaneous coaggregation, can be highlighted. Additionally, methods based on external stimuli such as the environmental conditions (pH and presence of ions), specific substrates, or the use of biopolymers are relevant [4,80], as demonstrated in Figure 2.

In fungus-induced pelletization, the process begins with the pre-culture of spores, which germinate and give rise to a mycelial network capable of forming compact granules. These granules act as capture nuclei, retaining microalgal cells through physical entanglement and biochemical interactions on the surface of the hyphae. The resulting structure tends to be highly dense and spherical, favoring rapid sedimentation and greater mechanical resistance. On the other hand, in microalgae-induced pelletization, the central role is played by the secretion of exopolymers (EPSs) or pH changes promoted by the microalgae themselves. EPSs function as an adhesive matrix, facilitating hyphal adhesion and the formation of heterogeneous aggregates. In these cases, the pellets exhibit a less compact morphology and greater structural variability [31,32].

In the context of microbial coaggregation, the role of neutralizing the surface electrical charges of cells is associated with the activation and reorganization of extracellular polymeric substances (EPSs) and intermolecular interactions involving proteins, polysaccharides, and other biopolymers. These processes promote the reduction of electrostatic repulsive forces, culminating in an efficient bioflocculation process. Consequently, external operational factors, such as pH variations, hydrodynamic agitation intensity, and substrate availability, directly influence the stability and dynamics of the system, modulating the efficiency of cell aggregation and creating space for the action of additional mechanisms involved in the formation and consolidation of microbial pellets [51].

In addition to their role in chemical adhesion, EPSs act as a three-dimensional encapsulating matrix, promoting the physical retention of microalgal cells within the hyphal network. This phenomenon is particularly important because many microalgae possess negatively charged cell surfaces due to the presence of carboxyl and phosphate groups in their cell walls. Under general conditions, these surface charges generate electrostatic repulsion among cells, thereby hindering spontaneous flocculation. The presence of EPSs mitigates this repulsive effect through a mechanism known as polymer bridging, whereby high-molecular-weight polymeric compounds simultaneously adsorb onto different cell surfaces and form physical bridges that connect microalgal cells to fungal hyphae. Furthermore, EPSs contribute to the microstructural organization of the aggregates by increasing the surface roughness of the hyphae. Rougher surfaces provide a larger specific area available for microalgal colonization, thereby increasing the number of contact sites between the interacting organisms. Consequently, this enhances the capture efficiency of suspended cells and improves pellet resistance to shear forces generated by aeration and mixing processes within the cultivation system [9].

Given the occurrence of metabolite–peptide communication profiles during microalgae–fungal interaction, recent studies have shown that enzymes such as laccases, cellulases (especially endoglucanases), and hemicellulases produced in co-culture systems, begin to act in a functionally integrated manner with extracellular polymeric substances (EPSs) [81]. These enzymes, associated with the extracellular matrix, play a central role in enhancing cell aggregation and show increased production and retention in EPSs, mainly due to signaling and response mechanisms induced by direct contact between cell walls, as well as changes in the physicochemical microenvironment established during interspecies interaction [82].

Among the microalgae–fungal consortia most reported in the literature, white-rot fungi such as *Trametes* and *Rigidoporus* stand out, as well as filamentous ascomycetes of the genera *Trichoderma*, *Penicillium* and *Aspergillus*, which are associated with widely studied microalgae such as *Chlorella*, *Tetradesmus*, and *Chlamydomonas*. Investigations aimed at comparing monoculture systems (*Trichoderma reesei* RUT-C30 and *Aspergillus niger*) and co-culture systems with mixed microalgae have shown a significant increase in both overall enzymatic activity and specific cellulase production. These results are attributed to the role of microalgae as suppliers of essential and trace elements (including micronutrients and growth factors) that act as metabolic inducers [82,83].

In substrate- or nutrient-stimulated pelletization, the role of carbon sources such as starch, glucose, and lactose stands out in intensifying the production of EPSs. EPSs are responsible for promoting the protective action of microbial cells when subjected to abiotic and biotic stresses (such as nutrient deficiency, radiation, and pH), presenting a homo- or heteropolymeric chemical composition with a high anionic configuration justified by the presence of sulfate, carboxylic, and phosphate groups [84,85], especially in fungi. Nutritional supplementation acts as a metabolic trigger, favoring the formation of larger and more resistant pellets. The concentration and feeding rate of these substrates are directly related to the degree of structural compaction and the ability of the pellets to withstand high hydrodynamic conditions [86].

A deeper exploration of the mechanistic investigation into aggregation between *Aspergillus fumigatus* and *Chlorella pyrenoidosa*, for example, demonstrated the production of N-acetyl-D-glucosamine (GlcNAc), a glucosamine derivative associated with the presence of glucose in the culture medium. This process was correlated with alterations in the surface topography of *Chlorella pyrenoidosa* cells, characterized by increased surface roughness, as observed by atomic force microscopy (AFM) analyses, which tends to increase the effective contact area and the intensity of short-range interactions. The results indicate that the conversion of glucose to GlcNAc occurs through the action of hydrolytic and degrading enzymes secreted by saprophytic fungi, which promote modifications in the microalgal cell wall. These structural alterations favor cell–hypha adhesion mechanisms, contributing to the intensification of aggregation and the stability of microalgae–fungal pellets [87].

Microalgae–fungal pelletization can be enhanced by modulating environmental parameters such as pH and ionic supplementation. In this sense, the surface matrix, when subjected to variations in parameters related to surface properties (charge/potential) and environmental conditions (ionic strength, pH, and multivalent ions), as well as agents that facilitate EPSs production and alter surface roughness, provides a greater understanding of the morphological formation process of the pellets and of stability analyses [88].

In the context of surface-engineered pelletization, In-na et al. (2020) [89] demonstrated that controlled modification of the cell–support interface plays a determining role in the morphology and functional performance of photosynthetic pellets. The authors developed microalgal biocomposites using vegetable sponge as a structural matrix coated with a latex-based polymeric binder, promoting the immobilization of *Chlorella vulgaris*, *Dunaliella salina*, and *Nannochloropsis oculata*. The process was carried out in a semi-continuous regime using inoculation corresponding to 5.0% (*w*/*v*) of cell biomass and 5.0% of binder solids. The surface engineering strategy favored the formation of compact and stable structures. These results demonstrate that operational parameters associated with binder composition, cell load, and culture conditions directly influence pellet morphology, strengthening surface-engineering pelletization as a promising approach for immobilized microalgal systems.

In addition to the method of pelletization, the development of performance evaluations and qualitative analysis of pellets regarding the operational parameters imposed in the process, as well as the identification of their respective influences, still requires intensive studies, because there is a range of variation when different strains are applied, showing different pelletization matrix results [90,91], as shown in Table 3.

As a response mechanism, Li et al. (2023) [92] proposed a hierarchical evaluation framework (integrating qualitative and quantitative criteria) for assessing pellet-based wastewater treatment systems using *Chlorella vulgaris* and *Aspergillus niger*. In this context, the term “weights” refers to the relative importance assigned to each evaluation parameter within the overall performance scoring system of algal–fungal granules. The adsorption saturation rate of mycelial granules on algae, cultivation/incubation time, and hydraulic shear stress (0.12–0.19) were identified as the most influential parameters. Conversely, variables such as light intensity and temperature exhibited lower relative weights (0.07–0.08). Complementary factors, including pH and salinity, were also incorporated into the evaluation, typically within operational ranges associated with optimal performance, such as moderate pH (≈5–6.5) and controlled temperature (≈30–37 °C), reinforcing their role in stabilizing system efficiency rather than acting as primary driving variables.

Furthermore, the development of mathematical models to predict microalgae–fungal flocculation significantly contributes to filling gaps in the understanding of the phenomena involved in the aggregation process. These models allow the description and quantification of fundamental structural and kinetic parameters, such as the density of microalgae–fungal granules and the consortium fixation constant ($K_{s}$) [28]. Assuming conditions of perfect spherical shape (fungal granules and microalgal cells), a non-growth phase during the interaction process, and no rupture of the pellet in the process, it is possible to obtain the number of cells for covering the fungal granule (N) (Equation (1)), the increase in mass of the alga–fungus granule due to algal fixation at time dt (Equation (2)), the constant associated with the progression of the radius of the algal–fungal granule over time (K) (Equation (3)), the flocculation rate constant (λ) (Equation (4)), the half-life of the flocculation reaction ($t_{1/2}$) (Equation (5)), and the density of the microalgae–fungal pellet (Equation (6)) [28].

(1) $$N=\frac{Surface\;area\;of\;fungal\;pellet}{Surface\;area\;of\;algae}=\frac{4\pi {r_{f}}^{2}}{4\pi {r_{a}}^{2}}$$

(2) $${Increase\;mass}_{pellet}=4\pi \rho N\left(\frac{{r_{a}}^{2}}{{r_{f}}^{2}}\right)Kt^{2}dt$$

(3) $$K={\left\{\frac{4}{3}\pi {r_{a}}^{3}nr_{f}\left(\frac{dv}{dx}\right)\frac{\rho_{a}}{\rho }\right\}}^{3}$$

(4) $$\lambda =\frac{K\rho_{f}}{\rho_{a}{r_{f}}^{2}r_{a}}$$

(5) $$t_{1/2}=\sqrt[{3}]{\frac{\ln2}{\lambda }}$$

(6) $${Density}_{pellet}=K_{s}\left(1-e^{-\lambda t^{3}}\right)$$

where $r_{f}$ and $r_{a}$ are the radii of a single fungal granule and microalgal cell, ρ, $\rho_{f}$ and $\rho_{a}$ are the densities of a microalgae-fungal pellet, single fungal granule and microalgal cell, respectively; n is the number of algal cells per unit volume, and $\frac{dv}{dx}$ corresponds to the velocity gradient.

The morphology of microalgae–fungal pellets is determined by multiple cellular aggregation pathways that reflect both intrinsic characteristics of the microorganisms and operational conditions imposed on the system. From the perspective of surface thermodynamics, the pelletization process involves critical parameters such as the contact angle of the formed granules, surface tension, and interfacial free energy, which govern aggregate stability. This process depends on the balance between electrostatic repulsion ($U_{ER}$) (Equation (7)), described by the zeta potential, and van der Waals attractive forces ($U_{VDW}$) (Equation (8)) [105] which can be combined as $U_{TOTAL}$ (Equation (9)), both of which are considered in the classical and extended Derjaguin–Landau–Verwey–Overbeek (DLVO) theory, ultimately determining whether adhesion or dispersion occurs [28,29].

(7) $$U_{ER}\left(D\right)=2\pi R\epsilon \psi_{S}^{2}ln\left[1+exp\left(-\kappa D\right)\right]$$

(8) $$U_{VDW}\left(D\right)=-\frac{AR}{12D}$$

(9) $$U_{TOTAL}\left(D\right)=U_{VDW}\left(D\right)+U_{ER}\left(D\right)$$

where *D* represents the separation distance between particles; A is the Hamaker constant; R corresponds to the cell radius; and ϵ denotes the dielectric constant of the medium. The parameters $\psi_{S}^{2}$ and κ represent the surface potential and the inverse Debye length, respectively.

It should be noted that the DLVO theory is based on the assumption of the total interaction energy between two spherical particles (Equation (9)) and has been widely applied to describe the stability of hydrophilic biocolloidal suspensions, such as microalgal systems, while considering the contribution of the Hamaker constant. However, the DLVO theory exhibits limitations in describing interactions involving microorganisms in aqueous environments, as it does not adequately account for certain surface-related phenomena. To overcome these limitations, Valin and Sutherland (1982) [106] proposed the extended DLVO (XDLVO) theory, which incorporates acid–base interactions and hydration forces into the original framework, thereby providing a more comprehensive and accurate description of microbial interactions in aqueous suspensions.

In an effort to develop a mathematical framework better suited to microbiological systems, Salim et al. (2013) [107] proposed a model based on the Smoluchowski aggregation theory, applied to a settling tank configured as a cascade of ideal mixers, to determine the particle production rate within a given size class *i* (Equation (10)). The model was validated using experimental data obtained from an autoflocculating microalgal strain (*Ettlia texensis*). Nevertheless, the application of the model requires the prior determination of strain-specific parameters, including density (ρ), minimum external porosity ($\varepsilon_{min}$) and individual cell diameter (d), as well as parameters associated with the experimental conditions, such as the initial number concentration, initial size distribution, and shear rate (G), which directly influence the collision efficiency (α) between particles.

(10) $$r_{i,z,t}=\frac{1}{2}\alpha \times \sum_{f=1}^{i-1}\frac{G}{6}\times {\left(d_{f}+d_{j}\right)}^{3}\times C_{f,z,t}\times C_{i-f,z,t}-\alpha \times \sum_{j=1}^{\infty }\frac{G}{6}\times {\left(d_{i}+d_{j}\right)}^{3}\times C_{i,z,t}\times C_{j,z,t}$$

where f, i, and j indicate the number of cells present in the flocs, and $C_{f}$, $C_{i}$, and $C_{j}$ represent the respective concentrations of flocs made up of f, i, and j cells (particle classes). The parameters $d_{f}$, $d_{i}$, and $d_{j}$ correspond to the diameters of the colliding particles, and; *z* and *t* represent the position and time of the particles considered. × is the multiplication sign.

Variations in the microalgae–fungal ratio during the pelletization process also directly impact the morphological characteristics (shape and size) and the final color of the pellet formed. According to studies of Liu et al. (2024) [97], the increase in microalgae concentration relative to the fungal fraction is inversely proportional to the size of the pellet formed, with results of 5–6 mm (1:1), 4–5 mm (5:1), 3–5 mm (10:1), and 1–2 mm (20:1). Furthermore, as the microalgal fraction increased, the incubation time required for complete pelletization decreased from 72 h (1:1) to 60 h and 48 h for the ratios of 5:1 and 10:1, respectively, and increased significantly to 96 h under the 20:1 condition. The optimal production point at the end of the studies was determined to be at a 10:1 ratio, given the characteristics of better growth and coloration (greenish), with the presence of microalgae not only on the mycelial surface but also in the internal region, indicating electrostatic neutralization of the pellet.

In microalgae–fungal pellet production, the agitation/aeration parameter is directly related to the frequency of collisions between cells and hyphae and mass transfer (O_2_/CO_2_ and nutrients). In aerated systems, experimental evidence shows that moderate aeration rates can maximize performance and biomass recovery, while excessive aeration tends to reduce efficiency due to microbial stress and, critically, by hindering the formation/stability of microalgae–fungal pellets (0.5–3.5 vvm as optimum in an intermediate range) [50]. Additionally, studies on fungal immobilization of microalgae indicate that low mechanical agitation regimes enhance adhesion efficiency by reducing shear forces, thus requiring careful choices between mechanical and pneumatic mixing by bubbling (air sparging) or airlift/bubble column configurations, frequently associated with milder shear profiles and hydrodynamics, which are favorable for co-pelletization [108,109].

Optimizing incubation time in the production of microalgae–fungal pellets was reported by Liu et al. (2024) [97], who applied *Chlorella vulgaris* and *Ganoderma lucidum*, and it was demonstrated that 56 h of incubation under agitation at 160 rpm significantly favored the performance of the consortium. Under these conditions, the maximum production of chlorophyll *a* (186.49 ± 17.81 µg·L^−1^) was observed after seven days of treatment of synthetic wastewater containing 10 µg·L^−1^ of tetracyclines. These values were substantially higher than those obtained in monoculture of *Chlorella vulgaris* under equivalent experimental conditions, where chlorophyll *a* production was approximately 100 µg·L^−1^.

## 4. Characterization Techniques for Microalgae–Fungal Pellets

The characterization strategies (physical, chemical, and biological) applied to microalgae–fungal pellets have advanced significantly in recent years, reflecting the increasing complexity of these hybrid systems and their relevance in environmental and industrial bioprocesses, as demonstrated in Table 4.

For the proper application of the different characterization techniques, it is important to emphasize the need for specific pre-treatment procedures for the pellets according to the requirements of each analytical method. In general, after pellet formation, the biomass is separated from the culture medium by sedimentation, filtration, or centrifugation and is subsequently subjected to successive washing steps using distilled water or buffered solutions, typically phosphate-buffered saline (PBS), to remove residual culture medium components, non-aggregated cells, and surface-adsorbed compounds [87]. Depending on the intended analysis, additional treatments may be required, including fixation with agents such as glutaraldehyde (1–2.5%) to preserve structural integrity during microscopic analyses [87], dispersion in electrolyte solutions (e.g., 1 mmol·L^−1^ KCl) to ensure stable and scientifically comparable electrokinetic measurements [80], or thermal treatment for cell lysis and enzyme denaturation during extracellular polymeric substances (EPSs) extraction and quantification, such as treatment with 0.05% NaOH under heating conditions [114].

### 4.1. Physical Characterization

The evaluation of the physical characteristics of microalgae–fungal pellets has generally been restricted to the application of conventional techniques, such as determination of dry mass and apparent density, optical microscopy, scanning electron microscopy (SEM), particle size analysis, and X-ray diffraction (XRD). It is worth noting that dry mass and density measurements are commonly performed and widely adopted as standard indicators of biomass production and pelletization efficiency. In contrast, particle size distribution analysis using laser-based techniques provides more detailed information on pellet structure and internal compaction. Together, these parameters are relevant for assessing sedimentation behavior [26].

Although these approaches provide relevant information on surface morphology, average granule size, and crystalline structural organization, they are still insufficient to comprehensively elucidate the architectural and functional complexity of these biological systems. In contrast, studies on the morphological engineering of fungal granules in bioprocesses to produce primary and secondary metabolites have incorporated more advanced analytical parameters, highlighting the importance of expanding physical characterization to optimize process performance and to better understand key aspects of microalgae–fungal consortia [25,26].

As an example, Dinius et al. (2024) [25] evaluated the formation of *Aspergillus niger* pellets under microparticle-added culture (MPEC) by combining confocal laser scanning microscopy (CLSM) and synchrotron radiation micro-computed tomography (SR-µCT), allowing for a non-destructive correlation of the three-dimensional architecture of the pellet (internal heterogeneity, incorporation/positioning of particles, and mycelial organization) with macromorphological attributes relevant to the bioprocess. This type of 3D characterization is particularly valuable because the pellet morphology, expressed by apparent density, fraction of dispersed mycelium, effective porosity, and thickness of the “hairy layer”, is simultaneously related to mass transfer, broth rheology, and, consequently, to the energy costs of agitation/aeration.

In mechanistic terms, dispersed/filamentous growth can accelerate biomass accumulation (greater interface area with the medium) but tends to increase viscosity and intensify non-Newtonian behavior, thereby increasing the energy demand for mixing and exacerbating transport limitations (substrates and O_2_), which directly impacts typical industrial platforms for fungi, as demonstrated by *Aspergillus* (enzymes and organic acids) [115,116].

Therefore, analogies for adhesion and stability studies of microalgae–fungal pellets, as well as consortium optimization, should not be limited to removal/production kinetics. It is necessary to integrate physicochemical and structural characterizations (CLSM for spatial distribution of biopolymers and SR-µCT for porosity/internal architecture) as morphological engineering tools to design pellets with mechanical robustness, favorable rheology, and internal diffusion compatible with bioprocess performance [25].

Another relevant physical characterization approach for pellets is the integrated use of scanning electron microscopy (SEM), transmission electron microscopy (TEM), and atomic force microscopy (AFM), which constitute a robust analytical framework for investigating the morphology, surface architecture, and internal organization of microalgal–fungal granules. Prior to analysis, pellets are typically washed to remove residual culture medium and fixed with glutaraldehyde to preserve structural integrity. For SEM, samples are freeze-dried, mounted on metallic stubs, coated with conductive materials, and analyzed to evaluate surface morphology, roughness, hyphal networks, and microalga–fungus interactions [87,94]. For TEM, fixed samples are dehydrated, embedded in resin, sectioned into ultrathin slices, and contrasted before imaging, enabling detailed visualization of intracellular structures, cell interfaces, and extracellular matrices within the pellet architecture [81].

The integrated application of these analytical tools has been widely employed to elucidate morphological, structural, and surface modifications resulting from microbial associations. An example is the study conducted by Bhattacharya et al. (2019) [87], which investigated biological granules formed through the co-cultivation of *Chlorella pyrenoidosa* and *Aspergillus fumigatus*. Using scanning electron microscopy (SEM), the authors observed significant changes in cell morphology and aggregate structural organization. These findings were further complemented by ultrastructural analyses performed through high-resolution transmission electron microscopy (TEM), allowing for a more detailed evaluation of the cellular modifications induced by microalga–fungus interactions.

However, electron microscopy techniques present limitations in obtaining quantitative parameters related to surface topography, and for this reason, atomic force microscopy (AFM) was additionally employed to characterize the aggregate surfaces at the nanometric scale. This approach enabled the acquisition of true three-dimensional images as well as the quantification of surface roughness and cellular dimensional changes. The results revealed an increase of approximately 14.5 nm in the root mean square (RMS) surface roughness and an increase of 11.3 nm in cell height compared with monocultures, indicating significant structural modifications associated with the co-aggregation process. Therefore, the combined use of SEM, TEM, and AFM proved to be an effective strategy for the comprehensive characterization of microalga–fungus interactions, providing complementary information on the morphology, ultrastructure, and surface properties of biological aggregates [87].

In a recent study, Chen et al. (2025) [110] evaluated the adsorptive potential of *Aspergillus niger* mycelial pellets functionalized with the metal–organic framework ZIF-67 (zeolitic imidazolate) using the Brunauer–Emmett–Teller (BET) method to characterize their textural properties. They observed that, during the adsorption of Congo red dye, the system exhibited a type IV adsorption–desorption isotherm characteristic of mesoporous materials, which is associated with the phenomenon of capillary condensation. These results demonstrate that the incorporation of metal–organic framework materials into the mycelial matrix significantly alters the specific surface area and pore distribution, increasing the efficiency of the pellet as a biosorbent.

Similarly, microalgae have been extensively investigated as natural adsorptive materials, especially in the removal of polycyclic aromatic hydrocarbons (PAHs), even in systems that are not necessarily pelletized. This performance is attributed to the chemical composition of the microalgal cell wall, which is rich in polar functional groups such as hydroxyls, phosphates, amino and carboxylic groups that favor electrostatic and complexation interactions with hydrophobic organic contaminants [51]. In this context, the conceptual integration between functionalized fungal pellets and microalgal biomass reinforces the relevance of physicochemical characterization techniques, such as BET, to understand and optimize adsorption mechanisms in microalga–fungus hybrid systems aimed at environmental applications.

The determination of the crystallinity index in microalgae–fungal pellets, as described by Gong et al. (2022) [26], is a relevant analytical approach for evaluating the structural organization of biomass and its influence on mechanical properties such as elasticity and structural strength. This index expresses the relative proportion between ordered (crystalline) regions, associated with well-organized polymeric structures, and disordered (amorphous) regions within the pellet matrix. The crystallinity index is typically obtained by X-ray diffraction (XRD), focusing on the angular range of 2θ between 18° and 22°, where the main diffraction signals of amorphous and crystalline phases are observed. In this context, I_22_ corresponds to the maximum diffraction intensity at approximately 22°, representing the crystalline fraction of the biomass, while I_18_ corresponds to the diffraction intensity around 18°, associated with the amorphous fraction. From these diffractograms, the index is calculated applying Equation (11), allowing the degree of structural ordering of the microalgae–fungal consortium to be inferred.

(11) $$CrI=\frac{I_{22}-I_{18}}{I_{22}}\cdot 100\%$$

During the pelletization process involving microalgae–fungal consortia, the release of hydrolytic enzymes, such as cellulases and xylanases, stands out and is associated with the partial degradation of structural polysaccharides (cellulose and starch) present on the surface of the microalgal biomass. The enzymatic action promoted by the fungal fraction contributes to rearrangements in the supramolecular organization of the biomass, resulting in significant alterations in the crystallinity index of the pellets when compared to monoculture systems. In this context, Gong et al. (2022) [26] reported crystallinity values of 50.88% for *Chlorella vulgaris*, 19.05% for *Ganoderma lucidum*, and 26.23% for microalgae–fungal pellets, highlighting the structural modulation promoted by the consortium. From an industrial perspective, the relative increase in the amorphous fraction in the hybrid pellets is particularly relevant, since less ordered structures tend to have greater accessibility to the functional groups of the biomass; it favors subsequent bioconversion steps, including fermentation and esterification processes, with direct implications for the efficiency of biofuel production, such as biodiesel, as well as other value-added products [117,118].

### 4.2. Chemical Characterization

The determination of the chemical composition of microalgae–fungal pellets, as well as that of the monocultures, is based on the identification and quantification of proteins, carbohydrates, lipids, and photosynthetic pigments (chlorophyll a and b, and carotenoids). This approach aims not only at a detailed characterization of the biomass obtained but also at understanding the possible routes for biotechnological use of the produced material. Additionally, the comparative analysis of these constituents allows for the elucidation of metabolic changes induced by the pelletization process, as well as the identification of interactions and synergistic relationships established between the microalgal and fungal fractions during co-cultivation [86].

Meanwhile, in the investigations conducted by Gangwar, Rautela and Kumar (2025) [44], the results of the biochemical composition, quantified in terms of dry cell weight (DCW), demonstrated that the microalgae–fungal ratio exerts a direct influence on the metabolic direction of the biomass formed. Treatments with exclusively microalgal participation showed a significant increase in lipid content (20.47 ± 0.38% DCW), in contrast to the fungal monoculture (9.50 ± 0.87% DCW) and the microalga–fungus co-culture in a 1:1 ratio (13.88 ± 0.73% DCW). Regarding protein accumulation, assays with a higher fungal fraction (1:2) significantly favored protein synthesis (26.59 ± 0.87% DCW), reflecting the structural and enzymatic contribution of the fungus in the consortium when compared to the 1:1 co-culture (21.93 ± 2.94% DCW) and the fungal monoculture (19.75 ± 1.20% DCW). In turn, carbohydrates were predominant in treatments with a lower microalgal ratio (1:2), reaching 22.93 ± 1.59% DCW, compared to the 1:1 co-culture (21.26 ± 0.36% DCW) and the microalgal monoculture (15.83 ± 0.82% DCW), possibly being associated with the accumulation of structural polysaccharides and extracellular polymeric substances. These findings reinforce that the pelletization process not only alters the morphological organization of the system but also modulates cellular metabolism, allowing the biomass composition to be directed according to the desired biotechnological application.

Furthermore, the application of analytical spectroscopy techniques for the characterization of microalgae–fungal pellets is widely adopted for the identification of substances through interactions between matter and radiation, thereby recording vibrational absorption bands characteristic of certain functional groups [119]. Infrared (IR) spectroscopy, including both dispersive instruments and Fourier transform infrared (FTIR) techniques, is widely applied to investigate surface and interfacial phenomena. Both approaches generate spectra within the mid-infrared region (4000–400 cm^−1^). However, they differ in their mode of data acquisition: dispersive IR systems record signals sequentially by isolating individual wavelengths, whereas FTIR instruments employ an interferometer to collect all wavelengths simultaneously, enabling faster analysis and improved signal-to-noise ratio [120].

When functional groups are evaluated using ATR-FTIR, a combination of Attenuated Total Reflectance (ATR) and Fourier Transform Infrared Spectroscopy (FTIR), applied to monoculture samples of the microalgae *Graesiella emersonii* and *Sphaeropleales* sp., to fungal granules of *Pleurotus pulmonarius*, and to microalga–fungus pellets, Miño et al. (2025) [95] identified fundamental relationships between specific bands and the pelletization process. Of particular note are the bands at 3413 cm^−1^ (N–H and O–H groups of amides), 2922 cm^−1^ (C–H stretching of alkanes), and 1745 cm^−1^ (C=O stretching typical of carboxylic acid derivatives), suggesting the direct participation of these functional groups in the physicochemical interactions responsible for pellet formation and reinforcing the need for further in-depth studies.

Complementarily, Guan et al. (2025) [86] observed, through FTIR spectra, the significant presence of proteins and polysaccharides in the EPSs composition. This was evidenced by bands associated with bending vibrations (i.e., with changes in bond angles) at 1550 cm^−1^ (N–H group of amide II), in addition to signals attributed to N–H and C=O groups characteristic of proteins. These findings corroborate the role of exopolysaccharides and proteins in mediating the interactions between fungal hyphae and microalgal cells during pelletization.

In parallel, Raman spectroscopy is configured as an inelastic scattering technique widely recommended for the analysis of symmetrical, nonpolar chemical groups (unlike FTIR, which is indicated for asymmetrical vibrations of polar groups such as O–H, N–H, and C=O). The complementary combination of these techniques significantly expands the capacity for structural characterization, allowing for more precise discrimination of the different chemical components present in the analyzed materials [121]. Furthermore, the incorporation of advanced computational tools stands out, including algorithms and libraries specialized in Python (Integrated Development Environment (IDE) software PyCharm (Community 2021.3), which expands the capacity for processing, extracting, and interpreting spectral information. Techniques such as PCA, PLS-DA, and machine learning-based methods are being applied to reduce dimensionality, identify spectral patterns, and improve discrimination between samples [122,123].

In evaluations involving different chemical states of compounds, X-ray photoelectron spectroscopy (XPS) stands out as a high-resolution surface analytical technique, particularly suitable for interface studies. This approach allows the identification and distinction between chemical species with bonds involving halogens, ionic salts, and neutral co-crystals, as well as the differentiation between these classes of compounds. Additionally, XPS has proven effective in detecting and characterizing nearly symmetrical hydrogen bonds, providing detailed information about the environment and oxidation state of the elements present on the surface of materials [124,125]. Applied to the characterization of microbial pellets based on Nie et al. (2022) [6], it was possible to identify carbon, oxygen, nitrogen, and phosphorus as the main chemical constituents of the elemental composition of both isolated microalgal and fungal fractions, as well as under cell aggregation conditions. This composition was confirmed by X-ray photoelectron spectroscopy (XPS), which highlighted peaks at 292.8 eV attributed to unsaturated conjugated systems associated with π–π* transitions, at 286.3 eV related to functional groups containing C–O and C–N, and at 284.85 eV corresponding to C–C and C–H bonds. A reduction in the intensity of these signals was also observed in samples subjected to microalga–fungus pelletization, indicating modifications in the chemical surface resulting from the cell aggregation process.

Furthermore, fluorescence spectroscopy analyses, characterized by high sensitivity, exhibit variations in the measurements performed and can thus be classified as Steady-State with measurements using continuous light, Time-Resolved with measurements by decay times, Synchronous Fluorescence Spectroscopy (SFS) with simultaneous measurement of excitation and emission monochromators in simple two-dimensionality, and Excitation Emission Spectroscopy (EEM/Fluorescence) with measurements of multiple emission spectra in relation to different excitation wavelengths in such a way as to generate three-dimensional intensity analyses with the obtaining of spectral profiles and analyte concentrations [126,127,128].

Regarding the detection of extracellular polymeric substances (EPSs) by fluorescence spectroscopy in microalga–fungus pellets, the presence of aromatic proteins, especially tyrosine and tryptophan residues, was observed, as reported by Nie et al. (2022) [6], as well as humic acids, according to Wang et al. (2021) [129]. EPSs associated with microalgae–fungal pellets are commonly extracted using mild alkaline and thermal treatments to promote the release of soluble polymers while minimizing structural degradation. Typically, pellets are treated with dilute NaOH solutions under controlled heating and agitation, followed by filtration or centrifugation to recover the soluble EPSs fraction [114]. These results indicate a higher productivity of protein-based compounds in microalga–fungus co-culture systems, as well as the intensification of the synthesis and release of new extracellular compounds by the microalgal fraction. This behavior is not observed under algal monoculture conditions, demonstrating that interspecific interactions in the aggregated system play a fundamental role in modulating the composition and complexity of EPSs. Applying this framework to the EMS/fluorescence results, the detection of tyrosine/tryptophan-like and humic-like fluorophores ceases to be merely evidence of the presence of EPSs and begins to support the interpretation that co-culture induces greater complexity and reactivity of EPSs, expanding functional sites capable of promoting structural cohesion and complexation/adsorption [7].

The identification and elucidation of the chemical composition of extracellular polymers (EPSs) produced during the pelletization process can be carried out using advanced techniques, such as ultra-high performance liquid chromatography coupled with high-resolution mass spectrometry, as demonstrated by Dulong et al. (2024) [112]. In the cultivation of the microalga *Glossomastix* sp., the authors reported the production of EPSs with a high degree of purity (approximately 86%), whose biochemical composition was predominantly made up of monosaccharides. Six-carbon methyl pentose sugars, such as rhamnose and fucose, stood out, in addition to galactose and smaller proportions of uronic acids, including galacturonic and glucuronic acids. Structurally, the EPSs presented recurrent units organized in Rha/Fuc–GalA/GlcA type chains, as well as domains enriched in Rha/Fuc or GalA/GlcA, evidencing high molecular complexity and relevant functional potential for cell adhesion and aggregation mechanisms. Furthermore, the macromolecular characteristics of EPSs, such as average molar mass distribution, polydispersity, and hydrodynamic conformation, can be determined by size exclusion chromatography (SEC), complementing the structural and functional characterization of these biopolymers [112].

The evaluation of the elemental composition of algal and fungal samples and their co-pelletization can reveal relevant compositional differences resulting from the co-cultivation process. Studies with *Trametes versicolor* and *Persinema* sp. indicated that the nitrogen (N) content showed a significant increase in the combined granules (15.24%) compared to isolated algal (5.27%) and fungal (13.63%) granules, indicating a greater incorporation of nitrogenous compounds, possibly related to the accumulation of proteins and extracellular polymeric substances (EPSs) during pelletization. The presence of phosphorus (P), sulfur (S), and inorganic ions such as K, Mg, and Cl was more pronounced in fungal and consortium granules, suggesting a greater capacity for adsorption and assimilation of mineral nutrients, a relevant aspect for applications in wastewater treatment [94].

The production of pellets by surface engineering methods, based on the application of sodium alginate (SA—5, 20 and 35 g·L^−1^), chitosan (CHI—20, 30 and 40 g·L^−1^) and polyvinyl alcohol (PVA—50, 100 and 150 g·L^−1^), all used as crosslinking agents, was evaluated in the formation of *Chlorella* sp. and *Aspergillus niger* granules. The analysis considered the zeta potential as an indicator of the surface properties of the pellets, observing a gradual increase in the surface potential compared to pellets without crosslinking agents (~−21 mV) and those containing SA (−13.05 mV, 20 g·L^−1^) and PVA (−16.36 mV, 100 g·L^−1^). In contrast, the application of CHI resulted in non-significant variations in the zeta potential, except at the concentration of 40 g·L^−1^, where a more pronounced reduction to −25.60 mV was observed [7]. Therefore, there is a general trend towards less negative zeta potential for SA and PVA, consistent with partial neutralization of the surface charge, while CHI does not follow this pattern and may even increase surface negativity at higher concentrations.

### 4.3. Biological Characterization

The analysis of biological characteristics in the production of microalga–fungus pellets has generally been conducted using integrated indicators of consortium performance and functionality, including growth kinetics and biomass productivity, enzymatic profile and activity associated with metabolism and/or contaminant degradation, cell viability and integrity throughout the operation (losses due to stress, diffusion limitation and shear) and physiological responses to operational conditions (pH, temperature, lighting, aeration, organic load and availability of N and P), parameters directly related to structural stability and performance in harvesting and wastewater treatment processes.

Evaluations of microalgae growth kinetics for pellet production were assessed by Paula et al. (2023) [113], given their direct influence on pellet formation/stability, such as cellular density available for adhesion to hyphae, metabolite and EPSs production, as well as changes in surface charge and physiology under stress—factors frequently associated with immobilization efficiency and pellet resistance during operation. Among the evaluations carried out, the following are most applied: volumetric biomass productivity (VPB), specific biomass productivity (SPB), specific growth rate (*µ*) and doubling time (t_d_), applying Equations (12)–(15), respectively, where N_T_ corresponds to the number of cells at the end of the logarithmic phase, N_0_ is equivalent to the number of cells at the beginning of the logarithmic phase, and T_T_ and T_0_ are the last and first days of the logarithmic phase, respectively.

(12) $$VPB\left(\frac{g}{L\cdot d}\right)=\frac{Dry\;biomass\;weigth}{Culture\;volume\cdot Culture\;time}$$

(13) $$SPB\left(d^{-1}\right)=\frac{VPB}{Biomass\;concentration}$$

(14) $$\mu \left(d^{-1}\right)=\frac{Ln(N_{T}/N_{0})}{T_{T}-T_{0}}$$

(15) $$t_{d}\left(d\right)=\frac{Ln(2)}{\mu }$$

Regarding cultivation conditions, the production and stability of microalga–fungus pellets are strongly modulated by the integrated responses of microorganisms to abiotic and biotic stresses. Abiotic factors, such as photoinhibition, salinity, pH, temperature, nutrient availability, and oxygen and CO_2_ gradients, directly influence cell growth, metabolic physiology, and cell surface properties, affecting bioavailability for aggregation and structural cohesion of the pellets [130,131]. Variations in these conditions can induce changes in the production of extracellular polymeric substances (EPSs), in surface charge, and in cell wall integrity—parameters closely related to microalgae–hyphae adhesion mechanisms and the mechanical strength of aggregates [7,132]. Thus, understanding cellular responses to multiple stresses is highly relevant for optimizing pelletization systems, allowing for the adjustment of operating conditions.

In this context, molecular research tools focused on analyzing gene expression associated with stress responses have gained increasing attention in scientific research, especially through quantitative PCR-based techniques such as qPCR (Quantitative Polymerase Chain Reaction) and RT-qPCR (Reverse Transcription Quantitative Polymerase Chain Reaction). These approaches allow for the obtaining of robust data on physiological responses to different stress conditions through the identification of changes in the expression of genes related to adaptive mechanisms [90,91].

Approaches focused on evaluating filamentous fungi, such as *Trichoderma atroviride*, subjected to osmotic (sorbitol and NaOH) and oxidative (H_2_O_2_ and menadione) stresses have demonstrated, through gene expression assays based on cDNA synthesis via reverse transcriptase followed by PCR reactions, direct alterations in cell wall integrity. These effects are associated with adaptive responses to osmotic stress mediated by the *nik1* gene, involved in the regulation and maintenance of cellular structure, as well as modifications resulting from the activation of the MAPK *Tmk3* pathway (mitogen-activated protein kinase), related to intracellular signaling. These alterations are reflected in relevant morphological and physiological changes, including variations in cellulose production and the conidiation process [130].

Analysis of enzymatic activity provides relevant insights into the metabolism of microalga–fungus pellets. Antioxidant enzymes and lipid peroxidation assays, when evaluated in the aggregation between *Chlorella vulgaris* and *Aspergillus oryzae* in synthetic wastewater, showed, through the biomarkers malondialdehyde (MDA), catalase (CAT), and superoxide dismutase (SOD), that under conditions of nutritional limitation, the antioxidant response was intensified as a defense system, as well as microalgae cultivation, in nitrogen-deficient media [30].

Regarding photosynthetic activity, the aggregation of these two microorganism fractions results in a decrease in the rate of photosynthesis due to the immobilization of microalgal cells. Photosynthetic pigments in microalgae–fungus pellets are commonly quantified following solvent extraction with organic solvents, particularly acetone, under refrigerated and dark conditions to prevent pigment degradation. After biomass separation, pigment extracts are analyzed by UV–V spectrophotometry at specific wavelengths corresponding to chlorophylls and carotenoids. This approach enables the determination of the photosynthetic capacity and physiological status of the microalgal component, providing important information regarding the effects of cultivation conditions and pelletization on cellular metabolism [133].

Therefore, investigative analyses are needed to understand optimal points suitable for balancing good microbial growth and, consequently, the production of microalga–fungus pellets in the system. Thus, measurements of chlorophyll *a* fluorescence emission based on the PSII protein complex (photosystem II) emerge as a control mechanism using a pulse amplitude modulation (PAM) fluorometer, aiming to quantify maximum ($F_{m}$) and minimum ($F_{0}$) fluorescence, variable fluorescence ($F_{v}$), and the steady-state fluorescence signal ($F_{s}$), thus enabling the acquisition of more in-depth parameters, such as the effective quantum yield of PSII (ΦPSII) (Equation (16)), referring to the real operational efficiency; the intrinsic efficiency of PSII (Equation ($F_{v}^{\prime }/F_{m}^{\prime }$) (17)); and the photochemical extinction coefficient (qP) (Equation (18)) [134,135,136].

(16) $$\Phi PSII=\frac{F_{m}^{\prime }-F_{S}}{F_{m}^{\prime }}$$

(17) $$F_{v}^{\prime }/F_{m}^{\prime }=\frac{F_{m}^{\prime }-F_{0}^{\prime }}{F_{m}^{\prime }}$$

(18) $$qP=\frac{F_{m}^{\prime }-F_{S}}{F_{m}^{\prime }-F_{0}}$$

where $F_{m}^{\prime }$ is the maximum fluorescence under light adaptation conditions, $F_{s}$ is the steady-state fluorescence signal, and $F_{0}^{\prime }$ is the minimum yield of stored fluorescence.

## 5. Conclusions and Future Perspectives

Following theoretical advancements in the formation of microalga–fungus pellets, this biotechnological system shows potential applicability based on its high structural robustness and metabolic complementarity between them, resulting in significant performance gains in environmental and industrial applications. The integration of advanced physicochemical characterization techniques, biological analyses, and predictive mathematical models has significantly broadened the understanding of the mechanisms involved in the formation, stability, and functionality of these aggregates. However, despite recent advances, further efforts are needed for methodological standardization, evaluation of strain-specific interactions, and scale-up of systems under realistic operating conditions. Further development of these integrated approaches will be fundamental to enabling the application of microalgae–fungal pellets in bioremediation processes, bioenergy production, and sustainable biorefineries.

Recent investigations have demonstrated that extracellular polymeric substances (EPSs), electrochemical surface interactions, and the architectural arrangement of fungal hyphae play a decisive role in the formation and maintenance of microalga–fungus pellets. Nevertheless, the individual contributions of these factors and the synergistic relationships established among them during aggregate development and long-term structural preservation are not yet fully elucidated.

Despite increasing evidence indicating the occurrence of biochemical communication, metabolic cross-feeding, and enzyme-mediated interactions within these consortia, the molecular networks regulating pellet assembly and maintenance remain insufficiently characterized. Future studies focused on deciphering these regulatory mechanisms may support the selection, adaptation, or engineering of microbial strains with superior aggregation capacity, enhanced structural stability, and improved operational performance.

From a theoretical standpoint, predictive approaches based on DLVO and XDLVO theories, together with aggregation kinetics models, have significantly contributed to the interpretation of pelletization phenomena. However, these models still employ simplified assumptions regarding cell morphology, extracellular matrix organization, and microbial growth behavior. Future developments should prioritize multiscale modeling strategies capable of simultaneously incorporating biological, physicochemical, hydrodynamic, and thermodynamic parameters. Furthermore, the integration of experimental datasets with machine learning algorithms and artificial intelligence techniques may improve the prediction of pellet morphology, aggregation dynamics, and stability under varying cultivation conditions.

Future research should also emphasize the development of harmonized and reproducible characterization protocols integrating morphological, physicochemical, biochemical, and functional analyses. The establishment of standardized methodologies would facilitate data comparison across different microbial consortia and cultivation systems while supporting the scale-up and industrial implementation of pellet-based technologies. In this regard, pellet characterization should evolve beyond a merely descriptive role and become a central tool for understanding the mechanisms of microbial aggregation, optimizing operational conditions, and designing advanced biostructured systems for applications in environmental biotechnology, resource recovery, carbon capture, and circular bioeconomy processes.

## Figures and Tables

**Table 2:** Values of biodiesel properties according to regulatory standards and different feedstocks.

Substrate	ν	*ρ*	SI		II	CI	OS	HHV	References
	(mm^2^·s^−1^)	(g·cm^−3^)	(g or mg KOH·g^−1^)		(g I_2_·100 g^−1^)	-	(h)	(MJ·kg^−1^)	
International Standards	1.9–6	-	≤0.80	-	-	>47	-	-	[58]
	3.5–5	0.86–0.90	≤0.50	-	≤120	>51	≥6	-	[59]
Commercial Diesel Petroleum	2.47–3.81	0.83–0.84	0.24	-	-	47.5	>12	42.18–49	[60,61,62]
*Mucorales* Fungi	3.5–4.7	0.86–0.88	-	202.5 ± 6	30.2–111.5	48–67.3	11.1–14.5	39.2–39.8	[53]
*Nannochloropsis* sp.	3.91	0.891	0.31	-	-	-	3	-	[63]
*Chlorella homosphaera*	4.7	0.871	0.37	-	-	54.31	-	19.08	[64]
*Chlorella protothecoides*	4.766	0.849	0.365	-	112.05	49.914	-	39.901	[65]
*Spirulina*	5.26	0.861	-	-	-	52.2	-	41	[66]
Rubber tree seeds	4.9	0.887	0.53	-	-	56.9	1.2	39.95	[67]
*Pongamia pinnata* seeds	4.1	0.76	0.41	-	-	-	-	≈31.93	[68]
*Maesa lanceolata* seeds	5.2	0.885	0.48	-	111.83	51.41	-	44.66	[69]
Residual safflower oil + Olive leaf (Antioxidant)	4.193	0.883	-	190	127	-	2.42	-	[70]
Residual safflower oil + Olive leaf (Antioxidant)	4.216	0.880	-	192	139	-	1.33	-	[70]
Waste margarine oil + Olive leaf (Antioxidant)	4.422	0.892	-	197	53	-	4.06	-	[70]

ν—Kinematic Viscosity, ρ—Density, SI—Saponification Index, II—Iodine Index, CI—Cetane Index, OS—Oxidative Stability, and HHV—Higher Heating Value.

**Table 3:** Optimized parameters in the formation of microalgae–fungal pellets.

Microbial Consortium	Incubation Time (d)		Fungus: Alga Ratio	pH	Temp. (°C)	Aeration (L·min^−1^)	Agitation (rpm)	Light Intensity (µmol·m^−2^·s^−1^)	References
	Alga	Fungus							
*Chlorella vulgaris Aspergillus niger*	3–5	3.75–4.5	-	5–6.5	33–37	-	155–165	54–67.5	[92]
*Picochlorum* sp. *Aspergillus niger*	20	1–2	-	7	28	0.5	150	40–47	[93]
*Persinema* sp. *Trametes versicolor*	-	-	1:2	6.5–8.5	30	-	130–150	60	[94]
*Graesiella emersonii Pleurotus pulmonarius*	14	5	-	-	28	-	100	50	[95]
*Tetradesmus obliquus Cunninghamella echinulata*	7	7	-	7.5	30	0.6	-	100	[96]
*Chlorella vulgaris Ganoderma lucidum*	7	7	1:1	-	25 ± 1	-	160	200	[97]
*Chlorella vulgaris Aspergillus flavus*	5	3	2.24:1	9.24	30	-	150	160	[5]
*Tetradesmus obliquus Penicillium gravinicasei*	7	~1	-	7.5	30	0.2–1.4	-	100	[50]
*Chlorella vulgaris Aspergillus oryzae*	-	2	10:1	-	30	-	130	-	[98]
*Chlorella vulgaris Ganoderma lucidum*	-	3	10:1	7.1	28	-	160	200	[45]
*Chlorella vulgaris Aspergillus niger*	3	3	-	5–9	27 ± 2	-	150	100	[99]
*Chlorella sorokiniana Aspergillus oryzae*	15	3	2.5:1	9	22 ± 1	-	150	70	[43]
*Chlorella pyrenoidosa Aspergillus oryzae*	3	2	-	3–7	30	-	180	-	[100]
*Chlorella vulgaris Aspergillus niger*	2	2	-	8	-	-	160	67.5	[101]
*Scenedesmus* sp. *Aspergillus tubingensis*	5	1	-	8	30 ± 2	-	120	≈50	[102]
*Chlorella* sp. *Ganoderma lucidum*	-	6	-	9	23	-	100	45	[103]
*Synechocystis* sp. *Aspergillus fumigatus*	3	3	-	5	25–30	-	160	-	[104]

**Table 4:** Current characterization techniques of microalgae–fungal pellets.

Typology	Characterization Techniques	Target Analysis	References
Physical	Dry mass, density and detachment rate	Analysis of yield, hydrodynamic behavior and mechanical stability of the pellet	[32]
	Laser particle size analysis	Size distribution by volume	[26]
	Microcomputed Tomography (µCT)	Porosity and internal channels	[27]
	Confocal Laser Scanning Microscopy (CLSM)	Three-dimensional visualizations of thin slices of granules	[25]
	Scanning Electron Microscopy (SEM)	Surface morphology, roughness, alga–hypha interface	[26]
	Transmission Electron Microscopy (TEM)	Morphology, size and internal structure of the pellet	[87]
	Atomic Force Microscopy (AFM)	Characterization of cellular topography	[87]
	X-ray Diffraction Spectroscopy (XRD)	Crystallization index	[26]
	Brunauer–Emmett–Teller (BET)	Surface area and pores	[110]
Chemical	Biochemical analyses (content of lipids, proteins, carbohydrates)	Compositional profile	[86]
	Fourier Transform Infrared Spectroscopy (FTIR)	Functional groups (carbohydrates, proteins, and lipids)	[86]
	Raman Spectroscopy	Chemical groups	[111]
	X-ray Photoelectron Spectroscopy (XPS)	Surface composition and chemical states	[6]
	Fluorescence Spectroscopy	EPSs composition	[6]
	Size Exclusion Chromatography (SEC)	Quantification and characterization of the molecular mass distribution of EPSs	[112]
	Zeta Potential	Surface charge	[7]
	Ultra-High Performance Liquid Chromatography Coupled to Mass Spectrometry	Identification of oligosaccharide sequences	[112]
	Energy Dispersive X-ray Spectroscopy (EDS/EDX)	Quantitative data and distribution of chemical elements (C, N, O, P, Mg, and Ca)	[94]
Biological	Cell death assay over time	Evaluation of microbial viability loss and physiological response to the environmental conditions of the assay	[113]
	PAM Fluorimetry (Pulse Amplitude Modulation)	Measuring photosynthetic activity	[26]
	qPCR/RT-qPCR	Analysis of stress gene expression	[90,91]
	Enzymatic tests	Evaluation of the activity of enzymes such as cellulase, laccase, and ATPase	[49]
	Bioremediation tests	Evaluation of the degradation and/or adsorption activity of organic matter, nutrients, heavy metals, and dyes, among others	[91]

## Data Availability

The original contributions presented in this study are included in the article. Further inquiries can be directed at the corresponding author.

## Abbreviations

The following abbreviations are used in this manuscript:

AFM	Atomic force microscopy
ATR	Attenuated total reflectance
ATR-FTIR	Attenuated total reflectance–Fourier transform infrared spectroscopy
ATPase	Adenosine triphosphatase
BET	Brunauer–Emmett–Teller method
C:N:P	Carbon-to-nitrogen-to-phosphorus ratio
CAT	Catalase
CLSM	Confocal laser scanning microscopy
COD	Chemical oxygen demand
COD:N:P	Chemical oxygen demand-to-nitrogen-to-phosphorus ratio
CO_2_	Carbon dioxide
CrI	Crystallinity index
DCW	Dry cell weight
DLVO	Derjaguin–Landau–Verwey–Overbeek theory
DRX/XRD	X-ray diffraction
EDS/EDX	Energy-dispersive X-ray spectroscopy
EEM	Excitation–emission matrix
EO	Oxidative stability
EPS	Extracellular polymeric substances
FTIR	Fourier transform infrared spectroscopy
F_0_	Minimum fluorescence
F_0_′	Minimum fluorescence in the light-adapted state
F_m_	Maximum fluorescence
F_m_′	Maximum fluorescence in the light-adapted state
F_v_	Variable fluorescence
F_s_	Steady-state fluorescence
GlcNAc	N-acetyl-D-glucosamine
IC	Cetane index/cetane number
II	Iodine value
IRF4	Interferon regulatory factor 4
IS	Saponification value
MAPK	Mitogen-activated protein kinase
MDA	Malondialdehyde
MEV/SEM	Scanning electron microscopy
MOF	Metal–organic framework
MPEC	Microparticle-enhanced cultivation
µ	Specific growth rate
µCT	Micro-computed tomography
NaOH	Sodium hydroxide
N_0_	Initial cell number (exponential phase)
N_T_	Final cell number (exponential phase)
PAM	Pulse-amplitude modulation
PCS	Higher heating value (Portuguese acronym retained)
pH	Potential of hydrogen
PSII	Photosystem II
PVA	Poly(vinyl alcohol)
qP	Photochemical quenching coefficient
qPCR	Quantitative polymerase chain reaction
RT-qPCR	Reverse transcription quantitative polymerase chain reaction
rpm	Revolutions per minute
SA	Sodium alginate
SEC	Size exclusion chromatography
SFS	Synchronous fluorescence spectroscopy
SOD	Superoxide dismutase
